# Chemogenetic Activation of Astrocytes in the Basolateral Amygdala Contributes to Fear Memory Formation by Modulating the Amygdala–Prefrontal Cortex Communication

**DOI:** 10.3390/ijms23116092

**Published:** 2022-05-29

**Authors:** Zhuogui Lei, Li Xie, Cheuk Hin Li, Yuk Yan Lam, Aruna Surendran Ramkrishnan, Zhongqi Fu, Xianlin Zeng, Shu Liu, Zafar Iqbal, Ying Li

**Affiliations:** 1 Department of Neuroscience, City University of Hong Kong, Hong Kong 999077, China; zhuogulei2-c@my.cityu.edu.hk (Z.L.); lixie7@cityu.edu.hk (L.X.); aramkrish3@cityu.edu.hk (A.S.R.); zhongqifu2@cityu.edu.hk (Z.F.); sliu93-c@my.cityu.edu.hk (S.L.); ziqbal2-c@my.cityu.edu.hk (Z.I.); 2 Department of Biomedical Sciences, City University of Hong Kong, Hong Kong 999077, China; chli57-c@my.cityu.edu.hk (C.H.L.); yukyanlam5-c@my.cityu.edu.hk (Y.Y.L.); xianlzeng2-c@my.cityu.edu.hk (X.Z.); 3 Centre for Regenerative Medicine and Health, Hong Kong Institute of Science & Innovation, Chinese Academy of Sciences, Hong Kong 999077, China; 4 Centre for Biosystems, Neuroscience, and Nanotechnology, City University of Hong Kong, Hong Kong 999077, China

**Keywords:** basolateral amygdala, medial prefrontal cortex, astrocytes, neurons, projection, chemogenetic, electrophysiology, fear conditioning, anxiety

## Abstract

The basolateral amygdala (BLA) is one of the key brain areas involved in aversive learning, especially fear memory formation. Studies of aversive learning in the BLA have largely focused on neuronal function, while the role of BLA astrocytes in aversive learning remains largely unknown. In this study, we manipulated the BLA astrocytes by expressing the Gq-coupled receptor hM3q and discovered that astrocytic Gq modulation during fear conditioning promoted auditorily cued fear memory but did not affect less stressful memory tasks or induce anxiety-like behavior. Moreover, chemogenetic activation of BLA astrocytes during memory retrieval had no effect on fear memory expression. In addition, astrocytic Gq activation increased c-Fos expression in the BLA and the medial prefrontal cortex (mPFC) during fear conditioning, but not in the home cage. Combining these results with retrograde virus tracing, we found that the activity of mPFC-projecting BLA neurons showed significant enhancement after astrocytic Gq activation during fear conditioning. Electrophysiology recordings showed that activating astrocytic Gq in the BLA promoted spike-field coherence and phase locking percentage, not only within the BLA but also between the BLA and the mPFC. Finally, direct chemogenetic activation of mPFC-projecting BLA neurons during fear conditioning enhanced cued fear memory. Taken together, our data suggest that astrocytes in the BLA may contribute to aversive learning by modulating amygdala–mPFC communication.

## 1. Introduction

Associative aversive memory formation, especially fear memory formation, is key to ensuring the survival of animals in a dynamic environment through adaptive behavioral responses [1]. Considerable evidence indicates that the basolateral amygdala (BLA) is crucial for the learning and memory expression of conditioned fear responses [1,2,3,4,5,6,7].

Investigations of associative memory have mainly focused on the neuronal mechanism while glial cells, such as the astrocytes, the most abundant cell in the brain, have seldom been researched [8]. However, there is now a growing body of evidence indicating that astrocytes actively contribute to synapse development, synaptic transmission, and neuronal excitability [9,10,11,12].

Recently, an elegant study demonstrated that chemogenetic activation of astrocytes in the hippocampus (HPC) can induce synaptic plasticity and enhance contextual fear memory formation [13]. Astrocytic Gi activation in the HPC during learning impaired remote contextual fear memory, accompanied by a suppression of anterior cingula cortex (ACC) neuronal activity during retrieval [14]. Moreover, a previous behavioral study revealed that chemogenetic activation of astrocytes in the central amygdala (CeM) enhanced within-session extinction (24 h after training) and decreased the expression of acquired fear responses [15]. In addition, Stehberg’s group demonstrated the crucial role of gliotransmitter release from astrocytes through Cx43 hemichannels in fear memory consolidation [16]. Furthermore, Ma’s group showed that Ras-related C3 botulinum toxin substrate 1 (Rac1) activity in BLA astrocytes is necessary for fear memory formation [17]. However, the further influence of BLA astrocytes in fear memory formation or expression remains to be elucidated.

Previous research has shown that astrocytes contribute to cortical slow oscillations [18,19,20], lending further support to the importance of astrocytes in network activity beyond tripartite synapses. Furthermore, astrocytes contribute to the generation of faster waves, such as theta (4–12 Hz) and slow gamma (30–50 Hz) [21]. Evidence shows that the prefrontal cortex and the BLA use theta (4–12 Hz) oscillations to communicate during and after fear conditioning [22,23]. In addition, astrocytic signaling plays an important role for theta synchronization and learning and memory in multiple brain regions [24]. It is reported that chemogenetic activation of astrocytes in the CeM specifically depresses excitatory inputs and enhances inhibitory inputs [15], pointing to its specific effects on neuronal circuits. The BLA projects to the prelimbic (PL) prefrontal cortex and the ventral hippocampus (vHPC), both of which are essential for the expression of auditory fear conditioning (FC) [25,26]. However, little is known about the influence of astrocytes in the BLA on the brain synchronization network.

To explore the role of astrocytes in the BLA in fear memory formation and expression, we employed the chemogenetic technique in this cell population. We found that astrocytic Gq modulation during learning resulted in an enhanced auditory cued fear memory performance, while astrocytic Gq modulation during memory recall had no effect on fear memory expression. Moreover, astrocytic Gq activation increased c-Fos expression in the BLA and the mPFC in fear conditioning, but not in the home cage. Combining these results with retrograde tracing, we found that astrocytic Gq modulation during fear learning enhanced BLA–mPFC recruitment. Electrophysiology recording showed that activating astrocyte Gq in the BLA promoted spike-field coherence and phase locking between the BLA and the mPFC. Finally, specific chemogenetic activation of BLA-to-mPFC projection during learning enhanced auditory cued fear memory.

This study may help to further reveal the mechanism of fear memory and may hold promise for the development of novel therapeutics that target astrocytes in the treatment of memory disorders.

## 2. Methods and Materials

### 2.1. Animal and Ethical Consideration

All the experimental work was carried out on adult male C57BL/6J mice (7–8 weeks old, Cat# JAX:000664). They were kept in cages with 24 h access to food chow and water. The animals were maintained in a holding room with a constant room temperature of 25 °C and a 12:12 h light-and-dark cycle. Animal studies were performed in accordance with the guidelines laid down by the Committee on the Use and Care of Animals, Department of Health, Govt. of Hong Kong SAR (Animals (Control of Experiments) Ordinance (Cap. 340), License to Conduct Experiments Ref: (20-17) in DH/HT&A/8/2/5 Pt. 1 and (20-112) in DH/HT&A/8/2/5 Pt. 1). Approvals for the “Ethical Review of Research Experiments involving Animal Subjects” were granted by the Animal Research Ethics Sub-Committee, City University of Hong Kong (Ref: A-0351 and A-0507).

### 2.2. Stereotactic Surgery and Virus Injection

The mice (P50–60 days) were deeply anesthetized using ketamine (100 mg/kg of body weight) and xylazine (8 mg/kg). The animals were placed on a stereotactic frame (RWD Instruments). A small volume of AAVs (adeno-associated viruses) was injected into the BLA bilaterally (AP, −1.36 mm; ML, ±3.24 mm; DV, −4.98 mm from bregma) using a modified microliter syringe (Hamilton) with a 32-gauge needle at a slow rate of 0.1 μL/min. For the retrograde tracing experiment, retro-AAV was injected into the mPFC bilaterally (AP, +1.90 mm; ML, ±0.25 mm; DV, −2.10 mm from bregma). After the injection was completed, the needle was left for an additional 10 min before it was slowly and completely withdrawn. After surgery, the animals were allowed to recover from anesthesia under a heat pad.

The following AAVs were used: AAV2/5-gfaABC1D-hM3q-mcherry (titer: 3.00 × 10^12^ v.g./mL, 0.15 μL, bilateral into BLA, Obio Technology); AAV2-retro-CaMKIIα-Cre (titer: 4.74 × 10^12^ v.g./mL, 0.15 μL, bilateral into mPFC, Taitool Bioscience); AAV8-hSyn-Dio-GFP (titer: 4.00 × 10^12^ v.g./mL, 0.15 μL, bilateral into BLA, Vigene Bioscience); AAV2/9-hSyn-Dio-hM3q-EYFP (titer: 5.00 × 10^12^ v.g./mL, 0.15 μL, bilateral into BLA, Vigene Bioscience). All viruses were aliquoted and stored at −80 °C until use.

### 2.3. Immunohistochemistry (IHC)

Three weeks after injection, the mice were transcardially perfused with cold PBS, followed by 4% paraformaldehyde (PFA) in PBS. The brains were extracted, postfixed overnight in 4% PFA at 4 °C, and cryoprotected in 30% sucrose in PBS. The brains were sectioned to a thickness of 0.03 mm using a sliding freezing microtome (Leica SM2010R) and preserved in a cryoprotectant 30% ethylene glycol in PBS. Free-floating sections were washed in PBS, incubated for 1 h in blocking solution (3% normal goat serum (NGS) and 0.3% Triton X-100 in PBS), and incubated overnight at 4 °C with primary antibodies (mouse anti-GFAP, Millipore, 1:500; rabbit anti c-Fos, Synaptic Systems, 1:500; mouse anti-NeuN, Cell Signaling Technology, 1:400; rabbit anti-S100β, abcam, 1:500) in 0.1% Triton and 3% NGS in PBS. The sections were then washed with PBS and incubated for 2 h at room temperature with secondary antibodies (goat anti-mouse, Alexa Fluor 488 and 405, abcam, 1:200; anti-rabbit, Alexa Fluor 405, 488, and 647, abcam, 1:200) in PBS. Finally, the sections were washed in PBS, mounted on slides, sealed with mounting medium (Fluoromount-G, eBioscience, San-Diego, CA, USA), and photographed using a Nikon Eclipse Ni-E upright fluorescence microscope and a Nikon A1HD25 confocal microscope (Tokyo, Japan). The images were acquired using identical gain and offset settings and analyzed with ImageJ and Adobe Photoshop software. Brain areas were manually outlined with reference to the “The mouse brain in stereotaxic coordinates, George Paxinos and Keith B. J. Franklin”. c-Fos-positive cells within the outlined mPFC (AP 1.54 to 2.10) and c-Fos-positive cells in the outlined BLA (AP −1.22 to −1.70) neurons (NeuN positive) were manually counted bilaterally by a trained observer blind to the treatment. The mean c-Fos density (c-Fos-positive neurons/mm^2^) was calculated as the numbers of c-Fos-positive neurons in one region divided by the area size of that region. Each group comprised 4 to 5 mice, with 5 sections per mouse for the BLA and the mPFC.

### 2.4. Behavioral Testing

#### 2.4.1. Fear Conditioning (FC)

The FC apparatus consisted of a conditioning box (30 × 30 × 50 cm), with a grid floor wired to a shock generator surrounded by an acoustic chamber and controlled by software (SuperMaze, Shanghai, China). Three weeks after injections, the mice were handled for three days and then habituated in the conditioning box for 2 min (Day 0). The mice underwent fear conditioning one day after habituation (Day 1, training). Specifically, the mice were placed in the conditioning box (box A) for 2 min, and a pure tone (3.0 kHz, 74 dB) was then sounded for 20 s, followed by a 1 s foot shock (0.5 mA). This procedure was then repeated twice with random intervals of about 80–120 s. The mice were returned to their home cages 30 s after the delivery of the third shock.

FC was assessed by a continuous measurement of freezing (complete immobility), the dominant behavioral fear response. Freezing was automatically measured throughout the testing trial by the SuperMaze tracking software.

On the next day, the mice performed a contextual memory recall test and an auditorily cued memory recall test (Day 2, test). Specifically, to test contextual memory, the mice were returned to the original conditioning box (box A), and the freezing of the mice was measured for 5 min. Then, 4 h later, to test auditorily cued memory, the mice were placed in a different context (a cylinder-shaped cage with stripes on the walls and a smooth floor, box B); 20 s tone (3 kHz) was then sounded three times with random intervals of about 80–120 s, and freezing during the tone was measured. Six days later, the auditorily cued memory recall test was performed again (Day 8, test). To further define the memory stage affected by BLA astrocytes, on day 9, we administered CNO (clozapine N-oxide) or saline in hM3q mice 30 min before the FC auditorily cued memory recall test.

#### 2.4.2. Non-Associative Place Recognition (NAPR)

The NAPR test was conducted in a round plastic arena (54 cm in diameter) [14]. In this task, the mice first explore a novel open field and are then expected to display decreased exploration of this now-familiar environment after re-exposure to the same arena. Specifically, the mice were placed in the center of the arena and allowed to freely explore for 5 min. Twenty-four hours later, the mice were placed again in the same arena for 5 min. The total exploration distance was measured using Anymaze (Stoelting Co., Wood Dale, IL, USA) tracking software, and the reduced exploration between the first and second exposures was calculated to show their memory for the place recognition.

#### 2.4.3. Open Field Test (OFT)

OFT testing was performed in a square enclosure (50 × 50 × 50 cm) to evaluate the animals’ locomotor activity and anxiety-like behavior. Thirty minutes after CNO/saline injection, the mice were placed in the OFT for five minutes. The open field was cleaned with 30% ethanol between each trial. The center of the OFT was defined as a square comprising 25% of the total area of the OFT (i.e., each length was 25 cm). The exploration distance, time, and entries of the center zone were measured using Anymaze (Stoelting Co., Wood Dale, IL, USA) tracking software.

#### 2.4.4. Elevated Zero Maze (EZM)

The EZM was made of grey plastic, 200 cm in circumference, comprised of four 50 cm sections (two opened and two closed) to evaluate the animals’ anxiety-like behavior. Thirty minutes after CNO/saline injection, the mice were placed in the EZM for five minutes. The maze was elevated 50 cm above the floor and had a path width of 4 cm with a 0.5 cm lip on each open section. The exploration distance, time, and entries of the open zone were measured using Anymaze (Stoelting Co., Wood Dale, IL, USA) tracking software.

#### 2.4.5. Chemogenetic Manipulation: CNO Administration

CNO was dissolved in DMSO and then diluted in 0.9% saline to yield a final DMSO concentration of 0.5%. The saline solution for control injections also consisted of 0.5% DMSO. A dose of 3 mg/kg CNO was intraperitoneally injected 30 min before the behavioral assays. The chosen doses of CNO did not induce any behavioral signs of seizure activity.

### 2.5. Electrophysiology Recordings

#### Electrode Implantation

The detailed procedures of the implantation of multi-channel recording electrodes have been described in our previous publications [10,27]. Briefly, after anesthetization, the skull was exposed, and two small holes (1–2 mm wide) were drilled above the ipsilateral BLA and the mPFC for electrode implantation. Five stainless bone screws were placed into the skull surrounding the surgical openings, and the dura mater was subsequently removed. Two 16-channel polyimide-insulated platinum/iridium micro-wire electrode arrays (4 × 4, electrode diameter = 25 μm; 250 μm spacing between each, impedance = 20–50 kΩ; Clunbury Scientific, Bloomfield Hills, MI, USA) were inserted into the mPFC (AP +1.90, ML −0.25, DV −2.10 mm from bregma) and the BLA (AP −1.36, ML ±3.24, DV −4.98 mm from bregma). Finally, a low-resistance 200 μm silver wire from each array was wrapped around one of the bone mounting screws for grounding. The recording electrodes were slowly inserted into the brain using a micropositioner until clear neuronal firings could be detected (OmniPlex system, Plexon, Dallas, TX, USA) in a majority of the recording channels.

### 2.6. Data Acquisition

Both the local field potential (LFP) and extracellular spike activities were recorded using a multiple-channel neural data acquisition system (Omniplex D, Plexon, Dallas, TX, USA). Spike signals were amplified (×1000), band-pass filtered (0.3–5 kHz, 4-pole Bessel), and sampled at 40 kHz. LFPs were amplified (×1000), band-pass filtered (0.05–200 Hz, 4-pole Bessel), and sampled at 1 kHz. To examine the role of astrocytic Gq manipulation on spike timing accuracy, 10 min’ data were recorded for each anesthetized mouse before CNO injection. Then, another 10 min’ data were recorded 30 min after the CNO injection.

### 2.7. Data Analyses

Data analyses, including spike sorting, spike-field coherence analysis, and analysis of phase locking of a single neuron to the theta oscillation were performed using a combination of tools in Matlab, Neuroexplorer, and Off-line Spike Sorter. Detailed procedures of multiple-channel recording of data and analyses have been described in our recent publications [10,27,28,29].

### 2.8. Quantification and Statistical Analysis

The sample sizes were chosen based on common practice in animal behavior. All data are presented as mean ± SEM, and the statistical analysis was carried out where appropriate using GraphPad Prism 9 (Graph Pad, San Diego, CA, USA). When only two groups were compared, unpaired Student’s *t*-test (two-tailed) was used. For other experiments, two-way ANOVA followed by Bonferroni’s post hoc test was used.

## 3. Results

### 3.1. Astrocytic Gq Pathway Modulation Promotes Auditorily Cued Fear Memory

**Figure 1:**
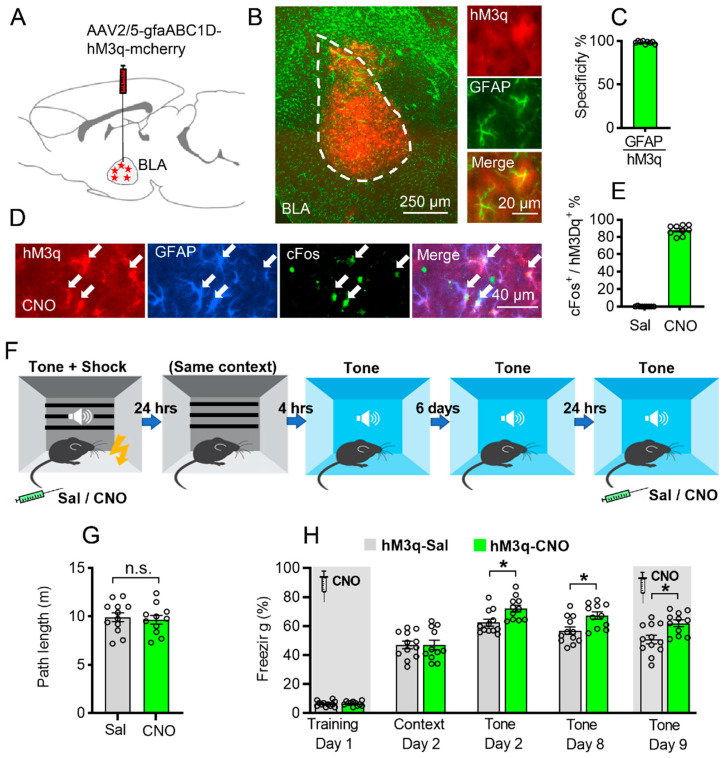
Astrocytic Gq activation in the BLA promotes auditorily cued fear memory formation. (**A**) Schematic picture showing AAV-gfaABC1D-hM3q-mcherry injection in the BLA bilaterally. (**B**) hM3q virus expression in the BLA (left panel); red is hM3q, green is GFAP staining, scale bar 250 µm. Magnified view of hM3q co-expressed with GFAP (right panel); scale bar 20 µm. (**C**) Cells with hM3q expression are highly co-labeled with GFAP (98.33 ± 0.5%, *n* = 3, 9 slices). (**D**,**E**) CNO administration to mice expressing hM3q (red) in BLA astrocytes resulted in a significant increase in c-Fos expression (green) in these astrocytes (white arrow), compared to saline-injected controls (*p* < 0.0001, *n* = 3 per groups, 9 slices per group; blue is GFAP staining, scale bar 40μm). (**F**) Schematic diagram of fear memory training and contextual/auditorily cued fear memory retrieval. (**G**) Path length of exploration in the conditioning cage after CNO or saline injection (*p* > 0.05, unpaired Student’s *t*-test, n.s. stand for no significance). (**H**) Chemogenetic activation of BLA astrocytes increased auditorily cued fear memory on day 2 (*p* = 0.0367), day 8 (*p* = 0.0424), but not contextual fear memory (*p* > 0.05) compared to saline group. On day 9, CNO injection before auditorily cued memory test still showed significant enhancement of fear memory compared to saline group (*p* = 0.0361) (Saline group *n* = 12, CNO group *n* = 11, * stands for *p* < 0.05, two-way ANOVA followed by Bonferroni’s post hoc test, main effect of Sal/CNO: F (1, 21) = 7.530, *p* = 0.0122). Data are presented as mean ± SEM.

Here, we hypothesize that BLA astrocytes can modulate fear memory. To specifically modulate the activity of BLA astrocytes, we injected AAV2/5-gfaABC1D-hM3q-mcherry virus into the BLA bilaterally, resulting in BLA-specific expression restricted to astrocytes (Figure 1A,B). The specificity of the virus was verified by co-staining with GFAP (98.33 ± 0.5%), S100β (98.00 ± 0.47%), and NeuN (0.55 ± 0.17%, Figure 1C and Figure S1).

Recent work has shown that hM3q activation of astrocytes induced NMDA-dependent de novo long-term potentiation in the HPC [13] and increased expression of the immediate early gene c-Fos in vivo [13,30]. To verify this effect ourselves, the mice were injected with CNO (3 mg/kg, i.p.), and their brains were collected 120 min later to be stained for c-Fos. As reported, CNO greatly increased c-Fos levels in the astrocytes of hM3q-expressing mice compared to saline-injected controls (87.89 ± 1.90%, *p* < 0.0001, Figure 1D,E).

To test the effect of astrocytic Gq pathway modulation on fear memory, the mice were injected bilaterally with AAV2/5-gfaABC1D-hM3q-mcherry into the BLA, and three weeks later, CNO (3 mg/kg, i.p.) was administered 30 min before fear conditioning (FC) training (Day 1). CNO application in GFAP::hM3q mice had no effect on the exploration of the conditioning cage (Figure 1G). Moreover, we found no difference in the freezing percentage during fear conditioning or in day 2 contextual memory retrieval between hM3q mice treated with CNO and those treated with saline (Figure 1H, left, *p* > 0.05). Remarkably, when the same mice were tested in another novel context with three-time tone 4 h later, those treated with CNO during training showed a significant increase in auditorily cued fear memory retrieval (*p* = 0.0367, two-way ANOVA followed by Bonferroni’s post hoc test, Figure 1H, middle). In addition, this significant memory enhancement by chemogenetic activation of BLA astrocytes during learning still existed in the day 8 auditory cue memory test (*p* = 0.0424, Figure 1H, right). To test whether Gq activation affects fear memory expression, we injected the CNO in the same mice before the day 9 auditory cue memory test and found that the CNO-treated mice still showed significant memory performance (*p* = 0.0361, Figure 1H, right), which was similar to that of the day 8 auditory cue test, indicating that the BLA astrocyte Gq activation may have no effect on memory expression.

To further verify that BLA astrocytic Gq activation does not have a direct effect on memory recall, we injected two new cohorts of mice with AAV2/5-gfaABC1D-hM3q-mcherry into the BLA, and i.p. injected CNO or saline 30 min before the auditorily cued fear memory test 24 h after conditioning. The CNO group showed no significant difference in auditorily cued freezing compared to the saline group (Figure S2). Taken together, it suggests that BLA astrocytic Gq activation enhanced fear memory during memory acquisition, and possibly during early consolidation, but not during memory retrieval.

To test the effect of astrocytic Gq pathway activation on memory acquisition in a less stressful task, we utilized the non-associative place recognition paradigm [14]. hM3D mice were injected with CNO or saline during memory acquisition and displayed a similar decline in exploration following exposure to the environment to which they were exposed 1 day or 8 days earlier (Figure S3A–F), indicating that astrocytic Gq activation has no effect on less stressful memory tasks. To confirm whether astrocytic Gq activation induced anxiety-like behavior, we employed the open field test and elevated zero maze (EZM). Chemogenetic activation of BLA astrocytes had no effect on the time spent in the center zone or in the open area of the EZM in hM3D animals with CNO compared to the saline group (Figure S3G–J). These findings suggest that astrocytic Gq pathway modulation in the BLA promoted cued fear memory but not contextual fear memory without anxiety effect.

### 3.2. Astrocytic Gq Activation Increases c-Fos Expression in the BLA and the mPFC in Fear Conditioning, but Not in the Home Cage

**Figure 2:**
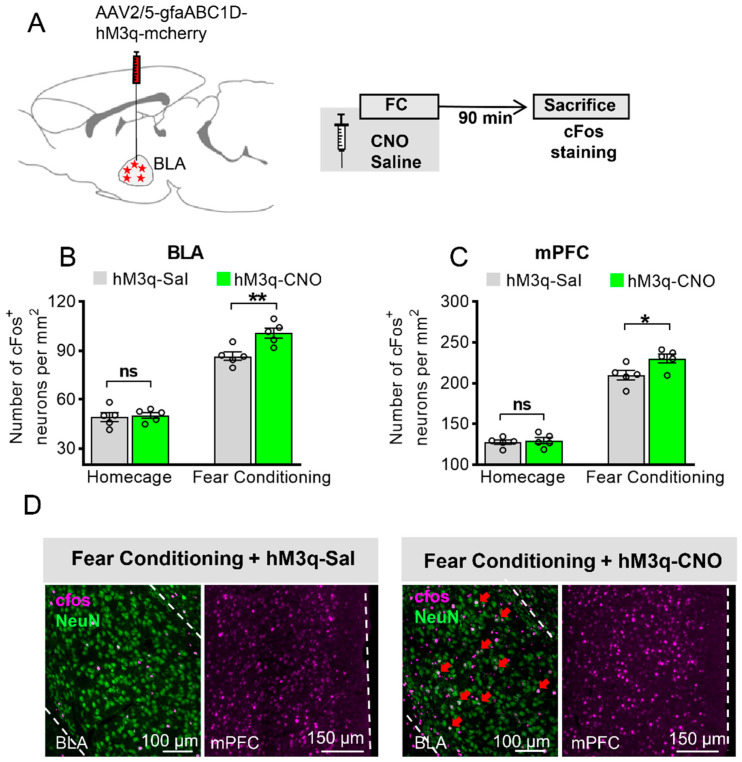
Astrocytic Gq activation increases c-Fos expression in the BLA and the mPFC in fear conditioning, but not in the home cage. (**A**) Schematic diagram for brain sample collection and c-Fos staining from mice injected with AAV2/5-gfaABC1D-hM3q-mcherry. (**B**) c-Fos level in BLA neurons of four-group mice expressing hM3q in their BLA astrocytes (*n* = 5 of each group, 5 slices for each mouse, two-way ANOVA with Bonferroni post hoc comparison test, main effect of Sal/CNO: F (1, 8) = 6.461, *p* = 0.0346, ‘homecage (HC) + hM3q-Sal’ versus ‘HC + hM3q-CNO’: *p* > 0.9999, ns stands for no significance, ‘fear conditioning (FC) + hM3q-Sal’ versus ‘FC + hM3q-CNO’: *p* = 0.0027, ** stands for *p* < 0.01, ns stands for no significance). (**C**) c-Fos level in mPFC neurons of four-group mice expressing hM3q in their BLA astrocytes (*n* = 5 of each group, two-way ANOVA with Bonferroni post hoc comparison test, main effect of Sal/CNO: F (1, 8) = 8.189, *p* = 0.0211, ‘HC + hM3q-Sal’ versus ‘HC + hM3q-CNO’: *p* > 0.9999, ns stands for no significance, ‘FC + hM3q-Sal’ versus ‘FC + hM3q-CNO’: *p* = 0.0127, * stands for *p* < 0.05). (**D**) Representative pictures of c-Fos (pink) expression in BLA neurons (NeuN, green) and mPFC under CNO or saline after fear conditioning, scale bar 100 μm, 150 μm. Red arrow shows the BLA neurons (NeuN) with c-Fos expression. All data are presented as mean ± SEM.

To gain insight into how BLA astrocytic Gq activation modulates neuronal activity, we activated BLA astrocytic Gq in vivo, either in home-caged mice or in mice that acquired FC, and then measured c-Fos levels as a marker for neuronal activity [13,31]. Another group of mice were bilaterally injected with AAV2/5-gfaABC1D-hM3q-mcherry into the BLA, and 3 weeks later, CNO or saline was administered in the home cage or 30 min before FC. Their brains were collected 90 min later and stained for c-Fos (Figure 2A). FC significantly increased the c-Fos levels in BLA neurons compared to home-caged mice (Figure 2B, two-way ANOVA with Bonferroni post hoc comparison test, F (1, 8) = 282.9, *p* < 0.0001). CNO administration to hM3q mice enhanced neuronal activity beyond the threshold for c-Fos expression compared to saline-injected mice when coupled with fear learning (Figure 2B,D, F (1, 8) = 6.461, *p* = 0.0346, *p* = 0.0027), but not in home-caged mice (Figure 2B, *p* > 0.9999). Moreover, in the same group of animals, we found that FC significantly increased the c-Fos levels in the mPFC compared to the home-caged group (Figure 2C,D, two-way ANOVA, F (1, 8) = 305.2, *p* < 0.0001). Furthermore, we found that hM3q mice that were given CNO and underwent FC showed a significant increase in c-Fos level in the mPFC compared to the saline group (Figure 2C,D, F (1, 8) = 8.189, *p* = 0.0211, *p* = 0.0117), indicating that astrocytic activation in the BLA during learning can enhance mPFC activity.

### 3.3. Astrocytic Gq Modulation Induced a Projection-Specific Enhancement of BLA–mPFC Neurons during Fear Learning

From our findings, BLA astrocytes modulate the BLA and mPFC activity during fear learning. We ask whether astrocytic Gq activation can selectively enhance the recruitment of mPFC-projecting BLA neurons. To directly address this issue, we labeled these mPFC-projecting BLA neurons by retrograde virus tracing, measured their activity during fear learning, and tested how they are affected by BLA astrocytic Gq activation.

**Figure 3:**
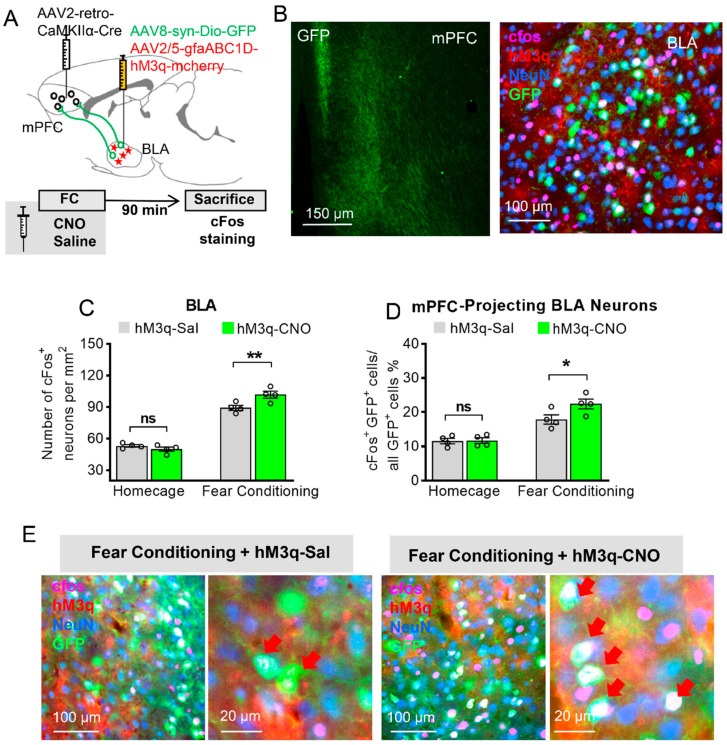
Astrocytic Gq modulation induces a projection-specific enhancement of BLA–mPFC neurons during fear learning. (**A**) Schematic diagram of targeting mPFC-projecting BLA neurons: AAV2-retro-CamkII-Cre was injected into the mPFC, and AAV8-syn-Dio–GFP together with AAV2/5-gfaABC1D-hM3q-mcherry was injected into the BLA. (**B**) Left: GFP-positive axons of BLA projection neurons are clearly visible in the mPFC. Right: mPFC-projecting BLA neurons (green) with c-Fos (pink) and NeuN (blue) staining, and hM3q (red) in BLA astrocytes. (**C**) c-Fos level in BLA neurons of four-group mice expressing GFP in mPFC-projecting BLA neurons and hM3q in their BLA astrocytes (*n* = 4 of each group, 5 slices for each mouse, two-way ANOVA with Bonferroni post hoc comparison test, main effect of Sal/CNO: F (1, 6) = 6.423, *p* = 0.0444, ‘homecage (HC) + hM3q-Sal’ versus ‘HC + hM3q-CNO’: *p* > 0.9999, ‘fear conditioning (FC) + hM3q-Sal’ versus ‘FC + hM3q-CNO’: *p* = 0.0055, ** stands for *p* < 0.01, ns stands for no significance). (**D**) Fear-conditioned mice injected with CNO showed increase in the percentage of mPFC-projecting BLA neurons that express c-Fos compared with FC mice injected with saline (*n* = 4 of each group, two-way ANOVA with Bonferroni post hoc comparison test, main effect of Sal/CNO: F (1, 6) = 7.424, *p* = 0.0344, ‘HC + hM3q-Sal’ versus ‘HC + hM3q-CNO’: *p* > 0.9999, ‘FC + hM3q-Sal’ versus ‘FC + hM3q-CNO’: *p* = 0.032, * stands for *p* < 0.05, ns stands for no significance). (**E**) Representative images of hM3q (red), c-Fos (pink), mPFC-projecting BLA neurons (green), and NeuN (Blue) in the BLA; red arrow shows the mPFC-projecting BLA neurons with c-Fos expression. All data are presented as mean ± SEM.

The mice were bilaterally injected with AAV2-retro-CaMKIIα-Cre into the mPFC and AAV8-syn-DIO–GFP together with AAV2/5-gfaABC1D-hM3q-mcherry into the BLA simultaneously (Figure 3A). The injection with these viruses allowed the GFP to be selectively expressed in the mPFC-projecting BLA neurons, and hM3q to be expressed in the BLA astrocytes (Figure 3B). Three weeks later, saline or CNO was i.p. injected in these mice 30 min before FC or in home-caged mice; then, these mice were sacrificed 90 min later. Similarly, during FC training, there was no significant difference between the CNO and saline groups in home-caged mice (*p* > 0.05), but CNO treatment under FC enhanced c-Fos expression in the BLA compared to the saline group under FC (Figure 3C, two-way ANOVA with Bonferroni post hoc comparison test, main effect of Sal/CNO: F (1, 6) = 6.423, *p* = 0.0444, ‘FC + hM3q-Sal’ versus ‘FC + hM3q-CNO’: *p* = 0.0055). When we specifically observe the sub-population of mPFC-projecting BLA neurons, these neurons were robustly activated during fear learning (Figure 3E). BLA astrocytic activation significantly enhanced the c-Fos expression in this sub-population of neurons under FC compared to the saline group under FC (Figure 3D, two-way ANOVA, F (1, 6) = 7.424, *p* = 0.0344, ‘FC + hM3q-Sal’ versus ‘FC + hM3q-CNO’: *p* = 0.032). Taken together, these data indicate that Gq manipulation in BLA astrocytes during fear learning may specifically enhance BLA-to-mPFC communication. However, the question of whether BLA astrocytic activation affects neurons projecting to other brain areas (e.g., nucleus accumbens (NAc) or vHPC) has not been addressed in this study and requires future investigation.

### 3.4. Activating Astrocytic Gq Pathway in BLA Facilitated Spike-Field Coherence and Phase Locking within BLA and between BLA and mPFC

**Figure 4:**
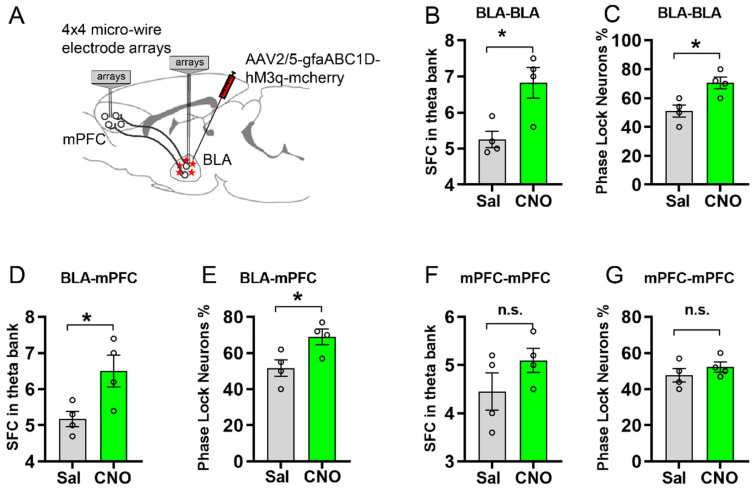
Electrophysiology recording showing that activation of astrocytic Gq in the BLA promotes spike-field coherence and phase locking within the BLA and between the BLA and the mPFC. (**A**) Schematic of the double-recording electrodes implanted in the BLA and the mPFC. (**B**,**C**) Quantification of spike-field coherence (SFC) within BLA in theta band and phase locking percentage within BLA between CNO and saline treatment (*n* = 4 per group, neurons number = 24, *p* = 0.0165, *p* = 0.0161, by unpaired Student’s *t*-test). (**D**,**E**) SFC between BLA and mPFC in theta band and phase locking percentage between BLA and mPFC with CNO treatment was increased compared with saline group (*n* = 4 per group, neurons number = 24, *p* = 0.0360, *p* = 0.0336, by unpaired Student’s *t*-test). (**F**,**G**) CNO application had no significant effect on SFC and phase locking neuron percentage within mPFC (*n* = 4 per group, neurons number = 24, *p* = 0.2066, *p* = 0.3688, by unpaired Student’s *t*-test). * stands for *p* < 0.05, n.s. stands for no significance All data are presented as mean ± SEM.

Previous evidence has suggested that the synchronization and oscillation of neuronal activity is essential to memory formation (Jutras & Buffalo, 2010). Recent studies have shown that Gq pathway activation in the HPC enhances neuronal activity through NMDA receptors [13,32], while Gq pathway activation in the CeM decreased neuron firing [15]. However, the contribution of astrocytes in BLA-to-mPFC spike-field coherence (SFC) and network synchronization has not been investigated. To address this issue, we recorded the spike and local field potential (LFP) in the BLA and the mPFC in anesthetized mice injected with AAV2/5-gfaABC1D-hM3q-mcherry into the BLA (Figure 4A). We obtained basal electrophysiological recordings of multi-unit activity under control conditions (over 30 min) and after an i.p. injection of CNO (3 mg/kg) or saline. In mice expressing hM3q in BLA astrocytes, CNO treatment significantly increased the SFC in the theta band within the BLA (*p* = 0.0165, Figure 4B) compared to the saline group. Moreover, the phase locking percentage of BLA neurons increased compared to the control group (*p* = 0.0161, Figure 4C). Furthermore, the SFC and phase locking percentage between the BLA and the mPFC were also significantly increased after astrocytic Gq pathway modulation in the BLA (*p* = 0.0360, *p* = 0.0336, Figure 4D,E). However, the SFC and phase locking percentage within the mPFC had no significant difference after astrocytic Gq pathway modulation in the BLA (Figure 4F,G), indicating that the astrocytic manipulation in the BLA may have no direct effect on the synchronization within the mPFC.

### 3.5. Specific Chemogenetic Activation of BLA-to-mPFC Projection during Learning Enhances Cued Fear Memory

**Figure 5:**
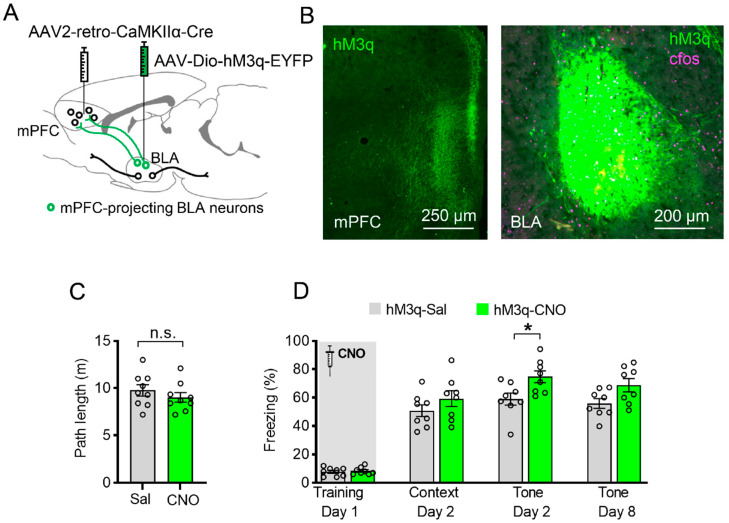
Specific chemogenetic activation of mPFC-projecting BLA neurons during learning enhances auditorily cued fear memory. (**A**) Schematic of the experiment: AAV2-retro-CaMKIIα-Cre was injected into the mPFC, and AAV2/9-syn-DIO–hM3q–EYFP was injected into the BLA. (**B**) Left: hM3q-positive axons (green) of BLA projection neurons are clearly visible in the mPFC. Right: hM3q (green) in BLA neurons projecting to the mPFC with c-Fos staining (pink). (**C**) Mice expressing hM3q in the mPFC-projecting BLA neurons were injected with either saline or CNO 30 min before FC training. CNO application before training had no significant effect on motor behavior (*p* = 0.3531, unpaired Student’s *t*-test, n.s. stands for no significance). (**D**) CNO application had no significant effect on contextual freezing level on day 2 (*p* > 0.4811) but promoted auditorily cued fear memory on day 2 (*p* = 0.0217) (Saline group *n* = 8, CNO group *n* = 8, * stands for *p* < 0.05, two-way ANOVA followed by Bonferroni’s post hoc test, main effect of Sal/CNO: F (1, 14) = 6.968, *p* = 0.0194). All data are presented as mean ± SEM.

To specifically manipulate BLA-to-mPFC neurons, mice were bilaterally injected with AAV2-retro-CaMKIIα-Cre into the mPFC and with a Cre-dependent hM3q virus (AAV2/9- DIO–hM3q–EYFP) into the BLA (Figure 5A). These viruses collectively induced the expression of hM3q–EYFP only in BLA neurons projecting to the mPFC (Figure 5B). Three weeks later, the mice were injected with saline or CNO 30 min before FC acquisition. CNO administration to mice expressing hM3q in mPFC-projecting BLA neurons had no effect on the exploration of the conditioning cage before shock administration (Figure 5C). When this group of animals were tested in the same context 1 day later, those treated with CNO during conditioning showed a similar contextual memory to the control (Figure 5D, left, *p* > 0.05). However, when the same mice were tested in a novel context with auditorily cued CS 4 h later, those with CNO treatment during conditioning demonstrated enhanced auditorily cued memory retrieval (Figure 5D, right, two-way ANOVA followed by Bonferroni’s post hoc test, F (1, 14) = 6.968, *p* = 0.0194, *p* = 0.0217). With this experiment, we provide evidence of the involvement of BLA-to-mPFC neurons in establishing auditorily cued memory during acquisition, as demonstrated by the effect of astrocytes on this process.

## 4. Discussion

Accumulating evidence has demonstrated the crucial role of astrocytes in the modulation of neuronal activity and plasticity, both of which are considered to be involved in learning and memory. Previous studies have reported that astrocytes in the hippocampus play an important role in contextual fear memory formation and remote memory formation, and that astrocytes in the CeM are necessary for fear memory expression using a chemogenetic tool. However, it remains unclear whether and how astrocytes in the BLA, one of the key brain regions for the regulation of fear memory, contributes to fear memory formation and expression. Here, we provide evidence that astrocyte activation in the BLA by chemogenetic Gq pathway manipulation promotes auditorily cued fear memory formation but has no effect on fear memory expression. In addition, we found that chemogenetic activation in BLA astrocytes during fear learning specifically enhanced BLA–mPFC communication. Using electrophysiology recordings, we further revealed that astrocytic Gq activation promoted spike-field coherence and phase locking between the BLA and the mPFC. Finally, we directly activated this projection using a chemogenetic technique to demonstrate its sufficiency for the enhancement of auditorily cued fear memory formation.

The role of astrocytes in learning and memory is still being debated. A growing body of evidence has shown the necessary role of astrocytes in memory, as interrupting astrocytic activity resulted in memory impairment [33,34,35,36]. Intriguingly, the engraftment of human astrocytes to the brain of neonatal immunodeficient mice led to an enhancement not only in LTP but also in learning ability during fear conditioning, Barnes maze testing, and object-location memory task [37]. However, the Gq pathway activation of astrocytes impaired long-term memory, while the deletion of astrocytic adenosine receptor A2A enhanced memory [38]. Moreover, chemogenetic or optogenetic Gq activation of HPC astrocytes during memory acquisition in mice can promote fear memory [13], while optogenetic activation of HPC astrocytes during memory consolidation in rats decreases fear memory [39]. These seemingly contradictory results suggest that astrocytes in different brain areas exhibit different roles in learning and memory. Our behavioral results are the first evidence, to our knowledge, to show that activating the astrocytic Gq pathway in the BLA resulted in enhanced auditorily cued fear memory formation but had no effect on memory expression. Xiao’s group found that optogenetic activation of BLA astrocytes can rescue anxiety-like behavior in stress mice [40], which seemingly contradicts our result (Figure S3). It is worth noting that Xiao’s study conducted an optogenetic application for 21 continuous days, which can induce anxiolytic effects. In contrast, we chemogenetically activated the mice once, and this does not induce any acute anxiogenic or anxiolytic effect. However, whether chronic chemogenetic manipulation of BLA astrocytes affects anxiety-like behavior needs to be further studied.

After astrocytic Gq activation in the BLA during fear conditioning, we found that the c-Fos level in the mPFC was significant increased compared to the control group (Figure 3C), while the c-Fos level in the dorsal or ventral HPC had no significant difference compared to control (data not shown). Moreover, the Klavir group found that optogenetic high-frequency stimulation of the BLA-to-mPFC synapses impaired cued fear memory consolidation but had no effect on contextual memory [41]. Furthermore, optogenetic activation of the BLA terminal in the mPFC was sufficient to promote defensive behavior during aversive stimuli, while inhibiting this terminal impaired the fear-associated memory [42]. Our chemogenetic data demonstrated the important role of BLA astrocytes and mPFC-projecting BLA neurons in fear memory formation. However, whether BLA astrocytic activation affects BLA neurons projecting to other brain regions (e.g., NAc or vHPC) has not been addressed in this study and requires future investigation.

Astrocytes interact with neuronal activity and release different neuroactive molecules, among which ATP (adenosine), glutamate, D-serine, and gamma-aminobutyric acid are the major gliotransmitters identified as regulators of synaptic transmission [12,43]. For instance, optogenetic activation of astrocytes in the hippocampus enhances the calcium signal in astrocytes and decreases fear memory through the adenosine A1 receptors [39]. How BLA astrocytes modulate local neuron and circuit, especially the PFC-projecting BLA neurons, needs to be further studied. In addition, our recent study showed that optogenetic activation of astrocytes facilitated anterior cingulate cortex spike-field coherence and phase locking in sham and visceral hypersensitivity rats via L-lactates [10]. Further investigation is required to determine whether L-lactate released by BLA astrocytes is involved in neuronal activity modulation and fear memory formation.

Several elegant studies suggest that astrocytes affect not only the local neuronal activity, but also the specific projections [14,15,44]. For example, astrocytic Gq pathway activation in the dorsomedial striatum can not only change the local neuron activity, but also modulate the activity of the downstream circuit, e.g., globus pallidus [44]. Moreover, astrocytic Gi pathway activation in the HPC during fear learning specifically decreased the communication of ACC-projecting HPC neurons [14]. In our study, we found that BLA astrocytic Gq activation may specifically enhance the BLA-to-mPFC recruitment. Moreover, direct activation of the BLA to the mPFC during fear learning can also enhance the auditorily cued fear memory (Figure 5), which is similar to the activation of BLA astrocytes (Figure 1).

Systemic brain activity is rhythmic; brain oscillations facilitate the synchronization of neurons and the formation of cell assemblies [45] that play an important role in memory formation [46]. Our previous works have revealed that phase locking and synchronization in the anterior cingulate cortex (ACC) and that between the ACC and the BLA are fundamental to the modulation of decision-making behavior in awake rats [10,27,28]. Facilitating ACC astrocytic activity by optogenetics rescued decision-making impairments in the chronic pain state and promoted decision-making behavior in normal rats [10]. Moreover, it is reported that astrocytic signaling is essential for the theta synchronization in multiple brain regions and learning and memory [24]. Evidence shows that the prefrontal cortex and the BLA use theta oscillations to communicate during aversive learning [22,23]. Moreover, our data demonstrate that astrocytes in the BLA contributed to fear memory by modulating the BLA–mPFC communication. Thus, it is important to investigate how astrocytes in the BLA may participate in this communication using electrophysiology. For the first time, we provide evidence that astrocytic Gq activation in the BLA can modulate BLA–mPFC synchronization by enhancing theta bank spike-field coherence and phase locking percentage between the BLA and the mPFC (Figure 4).

Though we can selectively activate the Gq pathway in BLA astrocytes by using chemogenetics, this approach comes with some limitations. First, this non-physiologic effect by chemogenetics could not mimic Gq signaling that occurs in physiological conditions. Second, the time resolution of chemogenetics is not enough to specifically manipulate astrocytes only during memory acquisition. Thus, more studies using a manipulation technique with a high time resolution, e.g., optogenetic, need to be conducted. Moreover, how astrocytic Gq signaling or related signaling is involved in fear conditioning needs to be further investigated.

In conclusion, we provide the first experimental characterization that chemogenetic activation of BLA astrocytes regulates auditorily cued fear memory formation but not memory expression. Our data also suggest that astrocytes in the BLA may contribute to fear memory formation by modulating BLA–mPFC communication. Our findings suggest a new therapeutic target for the treatment of memory-related disorders.

## Data Availability

The data supporting the current study have not been deposited to any public repository but are available from the corresponding author on request. This study did not generate code.

## Supplementary Materials

The following supporting information can be downloaded at: https://www.mdpi.com/article/10.3390/ijms23116092/s1.
