# Supplementary material for: Chemogenetic Activation of Astrocytes in the Basolateral Amygdala Contributes to Fear Memory Formation by Modulating the Amygdala–Prefrontal Cortex Communication
Source: Int J Mol Sci. 2022 May 29;23(11):6092. doi: 10.3390/ijms23116092 (PMC9181030; doi:10.3390/ijms23116092)
Supplement: Supplementary file 1 [file ijms-23-06092-s001.zip › ijms-1637557-supplementary.pdf]

SUPPLEMENTARY MATERIALS

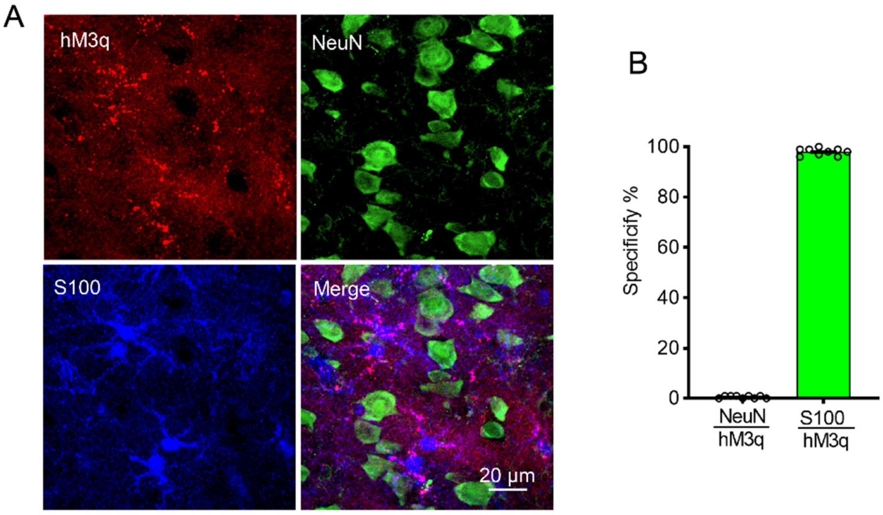

Figure S1. hM3q expression has high specificity. Related to Figure 1.

(**A**) hM3q (red) is co-expressed with S100 (blue) but not NeuN (green), scale bar 20 μm.

(**B**) Quantification of the percentage of co-expression level of NeuN/hM3q and S100/hM3q.

(n = 3 of each group, 9 slices per group, data are presented as mean ± SEM.)

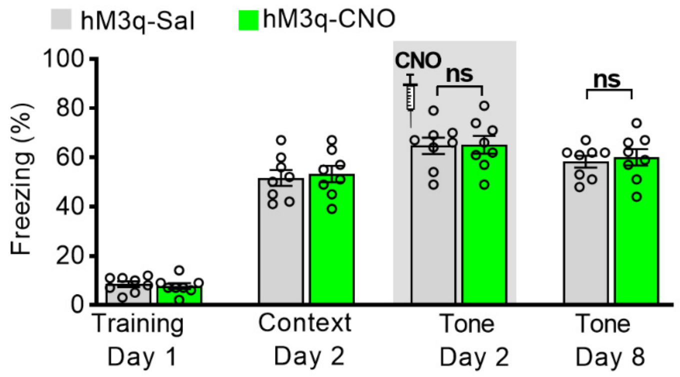

Figure S2. Astrocytic Gq activation in BLA does not have a direct effect on memory recall. Related to Figure 1.

Chemogenetic activation of astrocyte Gq in BLA during day auditory cued test on day 2 did not affect the cue fear memory on day 2 ($p > 0.999$) and day 8 ($p > 0.999$) (Saline group $n = 8$, CNO group $n = 8$, ns stands for no significance, two-way ANOVA followed by Bonferroni's post hoc test, main effect of Sal/CNO: $F(1, 14) = 0.1453$, $P = 0.7088$). Data are presented as mean ± SEM.

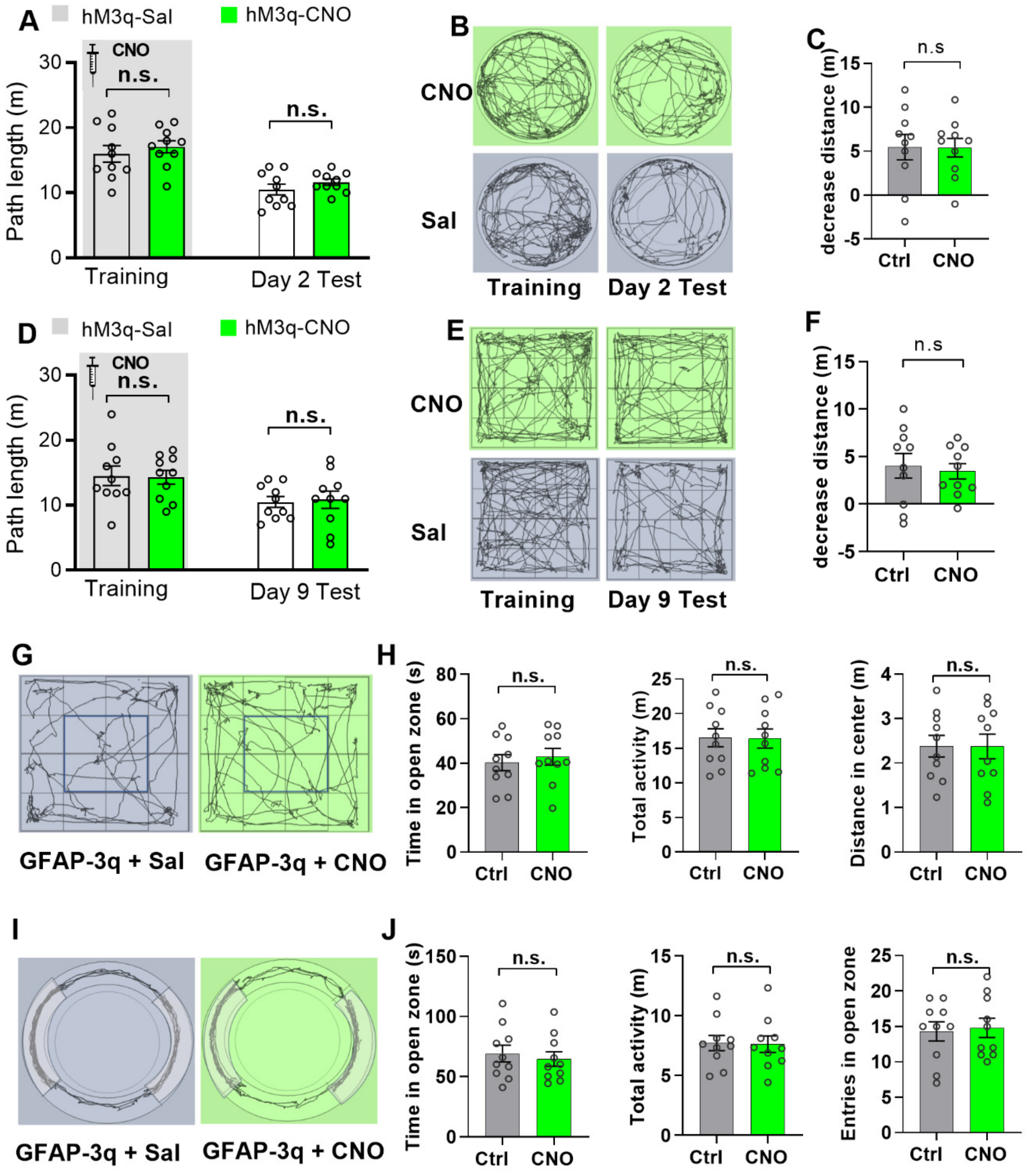

Figure S3. Astrocytic Gq activation in BLA did not affect non-aversive memory and did not induce anxiety-like behavior. Related to Figure 1.

(**A–F**) Astrocytic Gq activation did not affect the short-term (day 2 test) or long-term (day 9 test) association memory in the less-stress memory task.

(**G,H**) Chemogenetic astrocytic Gq activation did not affect the time in open zone (center zone), the total activity, or distance in center of OFT.

(**I,J**) Chemogenetic astrocytic Gq activation did not affect the time in open zone, the total activity, or entries in open zone in EZM (unpaired student t test, n = 10 in each group, data are presented as mean ± SEM).
